# Growth kinetics of the adsorbed layer of poly(bisphenol A carbonate) and its effect on the glass transition behavior in thin films†

**DOI:** 10.1039/d3ra02020g

**Published:** 2023-05-11

**Authors:** Hassan Omar, Gundula Hidde, Paulina Szymoniak, Andreas Hertwig, Andreas Schönhals

**Affiliations:** a Bundesanstalt für Materialforschung und -prüfung (BAM) Unter den Eichen 87 12205 Berlin Germany Andreas.Schoenhals@bam.de +49 30/8104-1617 +49 30/8104-3384

## Abstract

The glass transition behavior of thin films of poly(bisphenol A carbonate) (PBAC) was studied employing ellipsometry. The glass transition temperature increases with the reduction of the film thickness. This result is attributed to the formation of an adsorbed layer with a reduced mobility compared to bulk PBAC. Therefore, for the first time, the growth kinetics of the adsorbed layer of PBAC was investigated, prepared by leaching samples from a 200 nm thin film which were annealed for several times at three different temperatures. The thickness of each prepared adsorbed layer was measured by multiple scans using atomic force microscopy (AFM). Additionally, an unannealed sample was measured. Comparison of the measurements of the unannealed and the annealed samples provides proof of a pre-growth regime for all annealing temperatures which was not observed for other polymers. For the lowest annealing temperature after the pre-growth stage only a growth regime with a linear time dependence is observed. For higher annealing temperatures the growth kinetics changes from a linear to a logarithmic growth regime at a critical time. At the longest annealing times the films showed signs of dewetting where segments of the adsorbed film were removed from the substrate (dewetting by desorption). The dependence of the surface roughness of the PBAC surface on annealing time also confirmed that the films annealed at highest temperatures for the longest times desorbed from the substrate.

## Abstract 2

The growth kinetics of the adsorbed layer of poly(bisphenol A carbonate) was studied by a leaching approach and AFM.

## Graphical abstract

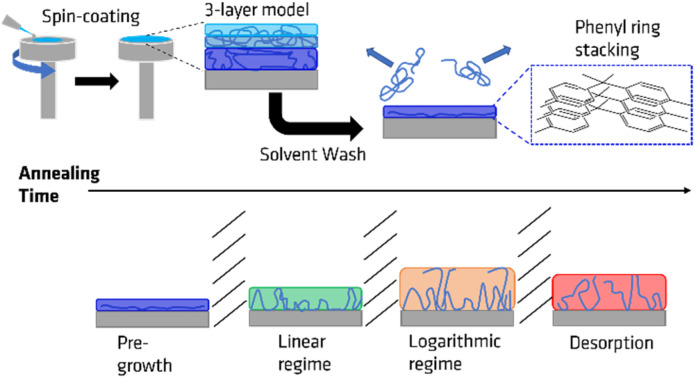

## Introduction

Thin polymer films play an important role in a variety of fields ranging from coatings to sensors and organic electronic devices due to their low cost and flexible production.^1–6^ Additionally, these films provide an ideal sample geometry to study the confinement effects of polymer segments at inorganic surfaces on their macroscopic properties.^7,8^ In studies of thin polymer films, properties such as structure, wetting, glassy dynamics, and the thermal glass transition (*T*_g_) were found to deviate from the bulk-like behavior.^6,9–14^ The thermal *T*_g_ is defined as the temperature at which the material falls out of thermodynamic equilibrium with decreasing temperature from a viscous liquid to a glassy solid. It is characterized by a slowing down of the molecular mobility of the segments.^15,16^ The investigation of *T*_g_ for thin films has led to contradictory results in previous studies, where an increase, a decrease, and no thickness dependence of *T*_g_ has been reported as the film thickness is reduced.^17–19^ From a general point of view, one must differentiate between the thermal *T*_g_ measured by so-called static methods such as calorimetry or ellipsometry and the dynamic *T*_g_ estimated at equilibrium in the frame of the linear response theory by relaxation spectroscopy.^1^ The thermal and the dynamic *T*_g_ can have different dependencies on film thickness. In the following, mainly results obtained by static methods will be considered. For polystyrene (PS), Keddie *et al.*^20^ found by ellipsometry that *T*_g_ decreases with decreasing film thickness independent of substrate. For other polymers such as poly(2-vinyl pyridine) (P2VP), polyvinylchloride (PVC), poly(methyl methacrylate) (PMMA) and poly(ethylene terephthalate) (PET) on a silica substrate an increase in the *T*_g_ with decreasing film thickness was reported.^6,21–23^

The dependence of the glass transition temperature on the film thickness has been shown to depend on interactions between the polymer segments and the substrate. Fryer *et al.*^24^ investigated the effect of the interfacial energy between a polymer film and the substrate and concluded that for a lower interaction energy the *T*_g_ of the thin film is decreased compared to the bulk value and is increased for higher interfacial energies. Xia *et al.*^25^ discuss a critical value of the interfacial energy for a specified film thickness. For values below the critical energy, *T*_g_ increases linearly with an increase in the interfacial energy and for values above, *T*_g_ becomes constant. However, using molecular dynamic (MD) simulations, Zhang *et al.* reported a decrease in *T*_g_ with decreasing film thickness regardless of the strength of the polymer–substrate interaction.^26^

For thin films on a non-attractive substrate, the decrease in *T*_g_ is discussed to originate from segments at the polymer/air interface having a higher molecular mobility compared to the bulk.^27^ This higher molecular mobility is due to missing segment/segment interactions. For any non-repulsive interaction between the polymer and the substrate, the polymer chains form an irreversibly adsorbed layer at the surface of the substrate.^28–30^ This adsorbed layer arises from specific chain/segment organization and/or packing at the substrate like for instance trains. The adsorption of the segments reduces the molecular mobility in the thin film which might result in an increase of *T*_g_. An investigation of PMMA and poly(vinyl acetate) (PVAc) adsorbed on silica revealed three regions in calorimetric signal of the samples having different thermal activities.^31^ The first region, at the polymer/substrate interface, is assigned to polymer segments in direct contact with the substrate forming loops, segments from a chain attached at two points, and trains,^32,33^ a series of segments in direct contact with the substrate. The next region, where polymer segments are located further away from the substrate is referred to a bulk-like layer. Lastly, the “mobile” region is at the polymer/air interface.^34^ Each of these idealized layers has a significant contribution to the glass transition behavior. For films the contribution of all these layers gives a complicated averaged *T*_g_ value.

The adsorbed layer at the substrate has gained a special interest due to the correlation between the number of adsorbed chains and the material properties.^35^ The adsorption of polymer segments to the substrate is discussed as irreversible due to the high energy barrier required for a simultaneous detachment of segments.^36^ The irreversible adsorption process is proposed by Guiselin.^37^ In that approach first macromolecules instantaneously adsorb onto an attractive surface from a polymer melt. Then the obtained thin film is rinsed with a good solvent and the loosely adsorbed chain are washed away. Only the tightly bounded chains remain at the substrate which are referred as “Guiselin brushes”.^38^ Finally, the so obtained adsorbed layer is then annealed at temperatures above the *T*_g_ of the bulk polymer to allow for further layer growth. Housmans *et al.* investigated the growth kinetics of the adsorbed layer of PS for different annealing times and temperatures. They showed that the adsorption process obeys a two-step kinetics with different time dependencies.^30^ At short times, the thickness of the adsorbed layer grows linearly with time by pinning segments directly to the substrate. This process leads to a crowding at the interface and after some time, the mechanism of growth changes to logarithmic one where the adsorbed layer grows by diffusion of segments through the existing layer on the cost of entropy (chain stretching).^30,34^ The crossover time, where the growth mechanism changes, has been found to dependent on both the molecular weight of the polymer and the annealing temperature. Reviews with a general discussion of the behavior of adsorbed layers including a discussion of the growth kinetics are given elsewhere.^33,39^

For this investigation, poly(bisphenol A carbonate) (PBAC) was chosen due to its high impact strength and thermal stability as well its importance in industrial applications, *i.e.*, precision optical parts and the active site in sensors.^40^ Additionally, there is little work that describes the confinement effects of PBAC and its relation to *T*_g_. Yin *et al.*^5^ studied the glass transition and dielectric behavior of thin films of PBAC capped between aluminum layers and found an increase in *T*_g_ with decreasing film thickness using dielectric dilatometry and dielectric relaxation spectroscopy. However, when the glass transition was studied using differential AC chip calorimetry, no thickness dependence of *T*_g_ was found down to a film thickness of 10 nm.^8^ Choi *et al.*^41^ studied the physical properties of PBAC and discovered that the Young's modulus and maximum stress decreased significantly for film thinner than 20 nm. Neutron scattering results on confined PBAC films pointed to a stronger localization of atoms in thin films supported on Si wafers.^42^ Therefore, obtaining an understanding into the thin film interactions for PBAC is of vital importance to correctly interpret the change in macroscopic properties. This work aims to investigate the influence of the adsorbed layer on the glass transition employing a combination of atomic force microscopy (AFM), and ellipsometry. The growth kinetics and the confinement effects will also be discussed in detail for the first time.

## Materials and methods

**Fig. 1:**
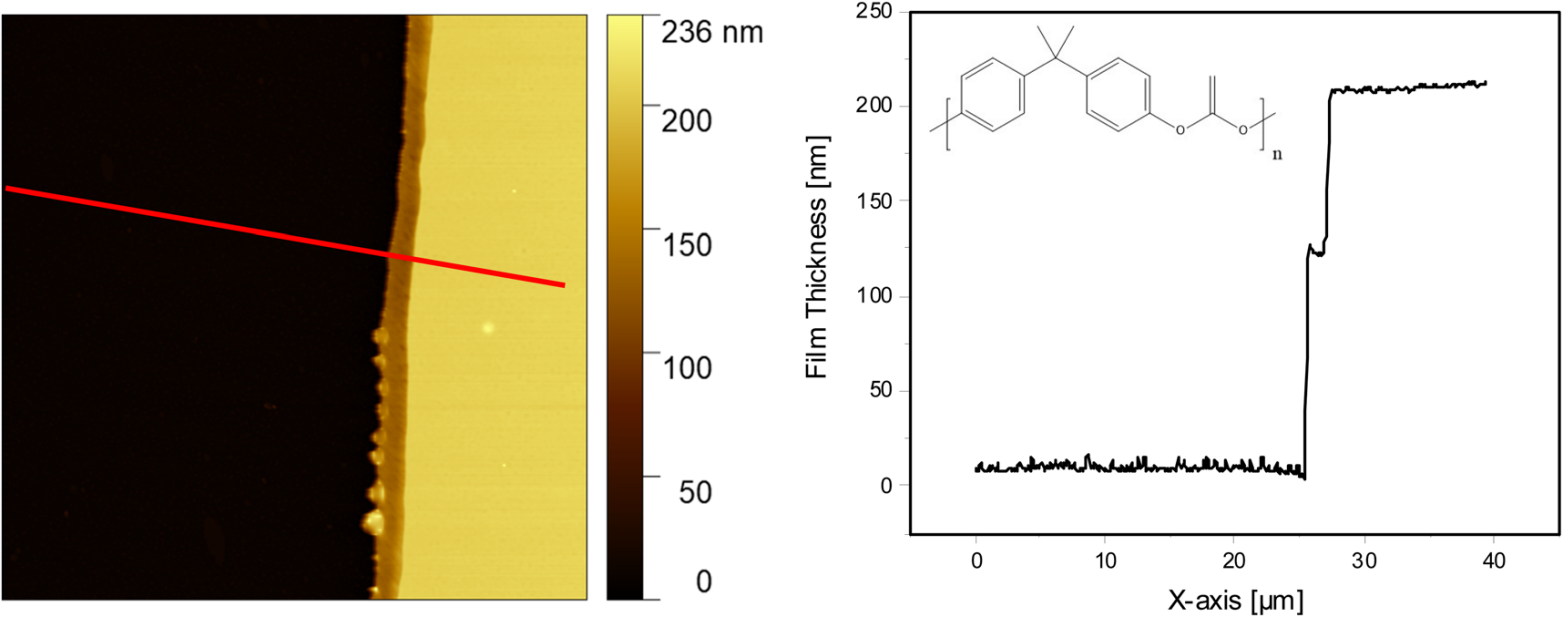
(Left) AFM image of the topography of a PBAC film with a thickness of 200 nm. (Right) the height profile taken from the indicated red line across the AFM topography image. The chemical structure of PBAC is given in the inset.

PBAC with a molecular weight (*M*_w_) of 24 400 g mol^−1^ and a PDI of 1.88 was purchased from Sigma Aldrich (Taufkirchen, Germany). It was not purified further. The samples were prepared by dissolving the PBAC pellets in dichloromethane (DCM) with various concentrations. The structure of PBAC is given in inset of Fig. 1.

### Differential scanning calorimetry (DSC)

Conventional DSC measurements were performed using a PerkinElmer DSC 8500 instrument (PerkinElmer, Waltham, MA, USA) with a heating/cooling rate of 10 K min^−1^ in the temperature range of 293–473 K. For the DSC measurements the samples were prepared by film casting a concentrated PBAC solution into a disk-shaped mold. Then the mold was placed it into a closed chamber where the atmosphere is saturated with the vapor of the solvent to allow for a slow evaporation of DCM. For a complete evaporation of the solvent, the film was then annealed for 72 h at 443 K in an oil-free vacuum. Nitrogen was used as a purge gas with a flow rate of 20 L min^−1^. Baseline corrections were performed prior to each run by measuring an empty pan and subtracting the measured heat flow from the data obtained for the sample. The second heating run was used for analysis.

### Preparation of the thin films

Diluted solutions of various concentrations were prepared to obtain thin films of PBAC with different thicknesses. Silica substrates (10 × 10 mm) were first washed with acetone to remove the protecting layer on the top surface. The substrates were then dried with compressed air and subsequently cleaned using super critical CO_2_ produced by a jet nozzle. Lastly, the substrate surface was activated in an oxygen plasma cleaner. 50 μL of PBAC solutions were pipetted onto the cleaned silica substrates. The samples were spincoated using the following parameters: 10 s with a rotational speed of 6500 rpm and an acceleration of 3000 r s^−2^ followed by 50 s, with 8000 rpm and 3000 r s^−2^. Different thicknesses were achieved by varying the concentration of the solution. The thin films were then annealed in an oil-free vacuum at *T* = 443 K (*T*_g_ + 30 K) for 72 h.

### Atomic force microscopy

AFM measurements were carried out using an MFP-3D origin atomic force microscope (Oxford Instruments, Abingdon, United Kingdom) in the tapping mode. For the measurements non-contact silicon SPM sensors (Nanosensors, Neuchatel, Switzerland) were used with a resonance frequency between 204 and 497 kHz. The scan rate used was 1 Hz with 512 points and lines per image. The thickness of each thin film was measured by making one continuous scratch through the film using a clean blade. Next, the difference between the glass surface and the top of the thin film was scanned. Multiple profiles were measured on each sample and the average value was taken as the film thickness. For processing the images, the software Gwyddion was used in the following steps. Firstly, any discontinuous lines or scars were removed and for bigger regions like artefacts where the image is hidden, a mask was applied. Next the rows were aligned using a polynomial degree and the option to exclude the masked regions. Lastly, after adjusting the scale and range for the color bar, the final image was obtained.

### Ellipsometry

A M-2000DI Ellipsometer (J.A. Wollam, Lincoln, NE, USA) was used with a multi-angle configuration to determine the thickness of the films and confirm the values obtained by AFM. The three angles of incidence used were 65°, 70°, and 75°. The wavelength range was from *λ* = 192 to 1697 nm (709 discrete wavelength points). Additionally, the temperature dependence of the thickness of each sample was measured where the temperature of the sample was controlled by a heating stage (INSTEC, Boulder, CO, USA). The angle of incidence for these measurements was 70°. The two ellipsometric quantities which are relevant for the measurements are the amplitude ratio of the polarized light (*ψ*) and the phase difference between the incoming and outcoming light (Δ).^43,44^

The spectra of the ellipsometric transfer data were analyzed by means of a model including the Si substrate and a native oxide layer with a fixed thickness of 2 nm. The datasets (dielectric functions) for this substrate are given by Herzinger *et al.*^45^ For the temperature scans discussed below a temperature-dependent variant for the substrate data are employed. The model was modified to include the sample temperature in every fit procedure when the temperature scan is analyzed. The temperature ramp was carried out form 25 °C (198 K) to 180 °C (453 K). The heating rate of the temperature scan was 2.5 K min^−1^. At the highest temperature the sample was isothermally held for 2 minutes to the check the stability of the whole system. The stability of the temperature was 0.1 K.

The dielectric function of PBAC was modelled in a two-step process. First, a pre-fit using a B-spline function was used to obtain starting values for the dielectric function.^46^ This pre-fit procedure was followed by describing the dielectric function polycarbonate in a general oscillator model.^43,47–49^ PBAC shows a structured absorption edge in the wave number range of UV light which required multiple peak functions to describe it. The initial spline fit was replaced by a proper oscillator model as the analysis method used for the temperature dependent measurements cannot be carried out by employing spline functions. The model parameters were optimized using multi-angle fits at room temperature. Due to the surface roughness of PBAC, the measured data might contain some artifacts caused by light scattering from the sample surface. Therefore, all fits are non-ideal to various degrees. Examples for a multi-angle fit and a fit during the temperature scan are given in the ESI in Fig. S1.† This figure shows that the employed model describes the experimental data well.

For analyzing the temperature dependent measurements, all fit parameters of the oscillator model were kept constant except the thickness of the PBAC film, *d*, and the offset of the real part of the complex dielectric function of PBAC, *ε*_∞_, at high frequencies. The analysis resulted in data for d(*t*) or d(*T*) by using the measured temperature of the sample at each point of the temperature scan where *t* is the measurement time and *T* the corresponding temperature.

### Solvent leaching

The adsorbed layer was obtained employing the solvent leaching approach by spin coating a 200 nm thick films onto a silica substrate and annealing it at three different temperatures for various times. For each annealing temperature a separate sample is prepared. The annealing and therefore the growing of the adsorbed layer was carried out in an oil-free vacuum. For details see ref. 30. It should be noted that the film for the leaching experiments must have a certain thickness otherwise no adsorbed layer will be formed and dewetting will take place.^34^ 200 nm was found to be the thickness to avoid these effects. After annealing, the films were then soaked in DCM for 20 minutes followed by a subsequent rinsing with DCM. Lastly, the films were then further annealed at the respective annealing temperature for 20 minutes to obtain the irreversibly adsorbed layer. The thickness of the irreversibly adsorbed layer was measured using the scratch technique described above.

## Results and discussion

Fig. 1 shows the AFM image (left side) and the height profile (right side) for the film obtained from spin-coating a 2 wt% PBAC solution onto the substrate and annealing the film for 72 h at *T*_g_ + 30 K. The thickness was determined to be 200 nm showing that the film topology is smooth. Table 1 gives the concentration of all considered solutions together with the obtained thicknesses. In addition to the thickness estimation by AFM, the thickness of each film was measured using ellipsometry employing a multi-angle configuration confirming the values obtained from AFM (see Fig. S2, ESI†). Both sets of experiments agree with each other and can be described by a common dependence.

**Table 1:** Thin film thicknesses obtained after spin-coating the respective solutions and annealing

Solution (wt%)	Thickness (nm)
2	200
1.5	140
1	90
0.8	75
0.7	69
0.5	55
0.3	45
0.2	26

The glass transition temperature for bulk PBAC was determined by fitting a sigmoidal function to the heat flow curve obtained from the DSC measurement and taking the derivative of the sigmoidal function (see Fig. S3†). The maximum position of the resulting peak was taken as the glass transition temperature with a value of 411 K, in agreement with values reported in literature.^2,8^

**Fig. 2:**
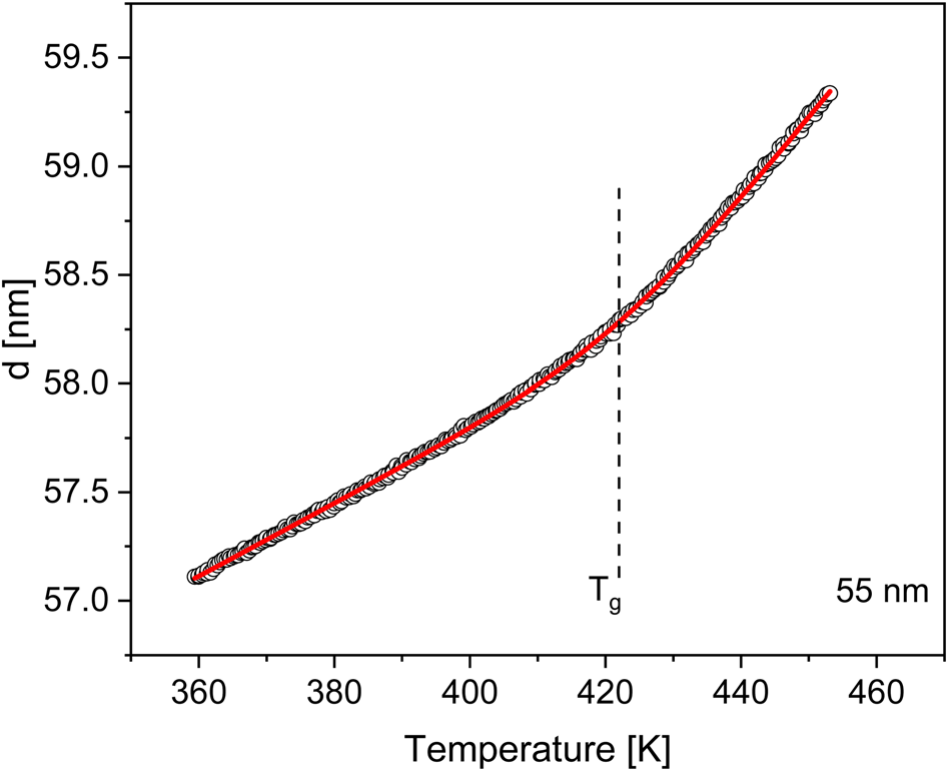
Temperature dependence of the thickness of a PBAC film with a thickness of 55 nm (at room temperature) during a heating run measured with ellipsometry. The red line is a fit of eqn (1). The dashed line represents the *T*_g_ estimated for this thin film sample.

**Fig. 3:**
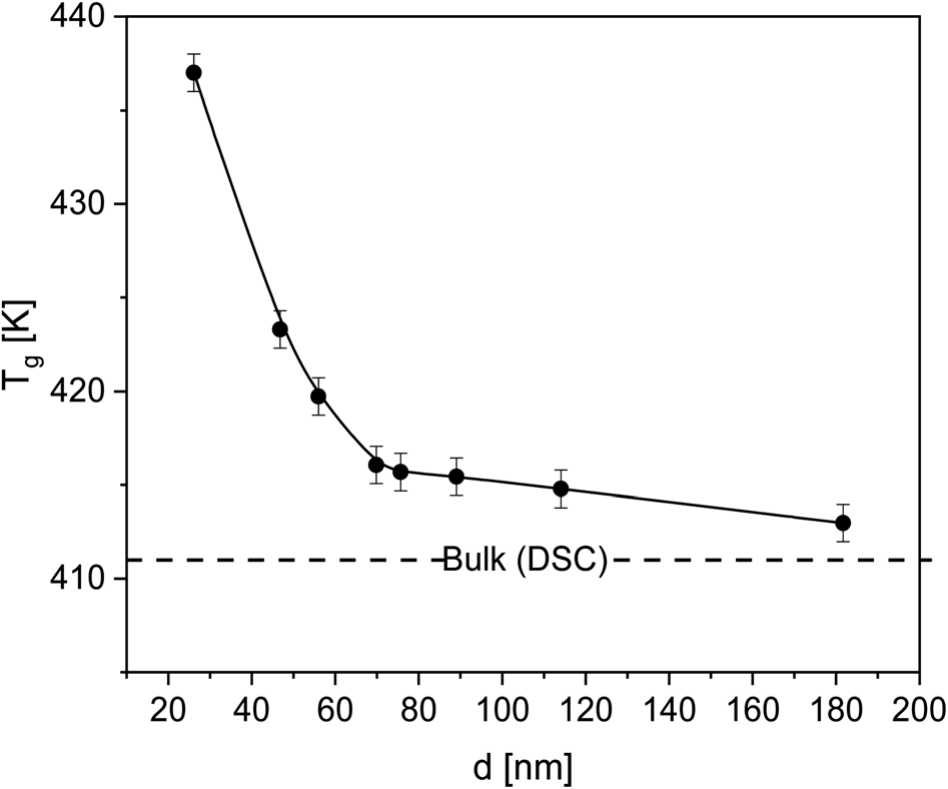
Glass transition temperature *T*_g_ of the thin films *versus* film thickness. The solid line is a guide to the eyes. The dashed line represents *T*_g_ of bulk PBAC obtained by DSC.

The *T*_g_ of the thin films of PBAC were estimated using ellipsometry by measuring the temperature dependence of the film thicknesses upon heating. As an example, the temperature dependent thickness for a film with a thickness of 55 nm at room temperature is depicted in Fig. 2. The glass transition temperature was estimated by fitting eqn (1) to the data, which was developed by Bittrich *et al.*^50^ to describe the non-linear change of the thermal expansion in the glass transition regime.1
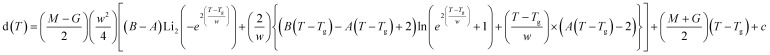
Here d(*T*) is the thickness, *w* denotes the width of the glass transition regime, *c* is the thickness of the film at the glass transition, and Li_2_ symbolizes the dilogarithm function. *G* and *A* describe the linear thermal expansion behavior in the glassy state where *M* and *B* models the same in the melt state. For the case *A* = *B* = 0, eqn (1) reduces to that of Dalnoki-Veress *et al.*^51^ However, it is worth mentioning that in contrast to the formula of Dalnoki-Veress *et al.*, eqn (1) has a theoretical basis. The estimated *T*_g_ values were plotted as a function of film thickness in Fig. 3, showing that *T*_g_ increases with decreasing film thickness. This result can be discussed considering the interactions between the polymer and the silica substrate to be strong. Further the result points to the formation of an immobilized layer at the substrate. Pham *et al.*^52^ investigated films of poly(tetramethylbisphenol A carbonate) (TMPC) on a silica substrate with ellipsometry and also reported an increase of *T*_g_ with decreasing film thickness. This was attributed also to strong interactions between the substrate and the TMPC segments due to hydrogen bonds formed between the hydroxyl groups of the substrate and the unshared electron pair of TMPC. For free-standing films of PBAC, Shamin *et al.*^53^ observed a decrease in *T*_g_ with decreasing film thickness, which has been commonly reported in the literature for other free-standing films such as for polystyrene.^20,22,51,54^ Therefore, the substrate–polymer interactions are the dominating contribution to the thickness dependence of the glass transition for PBAC thin films on Si. Yin *et al.*^8^ reported no change in the dynamic *T*_g_ as a function of the film thickness down to 10 nm, when measured by nanochip AC calorimetry where the film was spincoated on a SiO_2_ oxide layer. At the first glance this result contradicts the thickness dependence found in this investigation. However, the surface treatment of the silica substrate used here was not employed for the surface of the nanochip. The used surface treatment here may have allowed for enhanced interactions between the substrate and the PBAC segments. Also, the higher roughness of the nanochip might have played a role.

As stated above, the contribution of the free surface (polymer–air interface) was shown to decrease *T*_g_ due to the enhanced mobility of the segments which is due to missing segment–segment interactions. For the system investigated here, the adsorbed layer prevailed any contribution from the free surface and might serve as direct proof of the adsorption of PBAC on silica. The minor effect of the free surface effect due to irreversible adsorption has also been reported for various other systems, poly(4-*tert*-butylstyrene),^55^ polystyrene,^56,57^ poly(2-vinylpyridine).^57^ Xu *et al.* investigated the molecular dynamics of poly(ethylene terephthalate) films^58^ and found that the distance at which the substrate effect can influence the *T*_g_ behavior, the propagation distance, increases with decreasing thickness causing a reduced segmental mobility. The free surface effect was only observed when the segments at the polymer/air interface were unperturbed by the substrate. Also, the effect of the chain conformation at the substrate plays a vital role in the glass transition of thin films. Zuo *et al.*^59^ showed that adsorbed chains having larger loops led to further propagation of interfacial effects in the bulk-like layer due to the entanglements between these loops and the loose chains in the bulk-like layer. This region is denoted as overlap region. The thickness of this overlap region increases with an increase in the thickness of the adsorbed layer and suppresses the segmental mobility and vitrification.^60^

For PBAC films with thicknesses below 28 nm, from the measured ellipsometry data no glass transition could be estimated as direct consequence of the small changes in the ellipsometric quantities upon heating.^44^ Both optical parameters, and therefore the film thickness, are influenced by thermal expansion of the sample. Specifically, the thermal expansion coefficient (*α*) and the change in refractive index (*n*) with temperature must be considered. For PBAC low values for both coefficients compared to other polymers,^61^ 1.7 × 10^−4^ K^−1^ and −0.9 × 10^−4^ K^−1^ respectively, have been reported. Therefore, with the reduction in the film thickness the measured changes in the refractive index during heating become comparable to the uncertainty of the measurement. Additionally, ellipsometry requires a specific model for fitting the raw data using assumptions about the layers which can become inaccurate for thinner films.^62,63^ Deviations from the assumed layer properties, like specific orientations or ordering of segments, would also have a greater effect on the analysis of the data measured for thinner films. Moreover, a surface induced crystallization could be not completely excluded for film thicknesses below 28 nm.

For PBAC, the segments might display a preferential orientation after spin-coating onto an attractive substrate. Specifically, the phenyl rings in the polymer chain backbone can adopt a parallel orientation, to the substrate due to polymer–substrate interactions. Myers *et al.*^64^ investigated the effect of the solvent on the phenyl ring ordering for several polymers using sum frequency generation (SFG). The polymers studied had phenyl rings in the backbone (PBAC and polysulfone (PSF)) and in the side chains (poly(phenyl methacrylate) (PPM), poly(benzyl methacrylate) (PBM) and poly(methyl phenyl siloxane) (PMPS)). It was found that for a non-aromatic solvent the phenyl rings of PBAC might have a preferred parallel orientation to the surface when spin-coated from a 2 wt% solution. For other concentrations no preferential orientation was observed. Therefore, a preferential orientation of the phenyl rings cannot be excluded and might complicate the analysis of the ellipsometric measurements for films thinner than 28 nm.

**Fig. 4:**
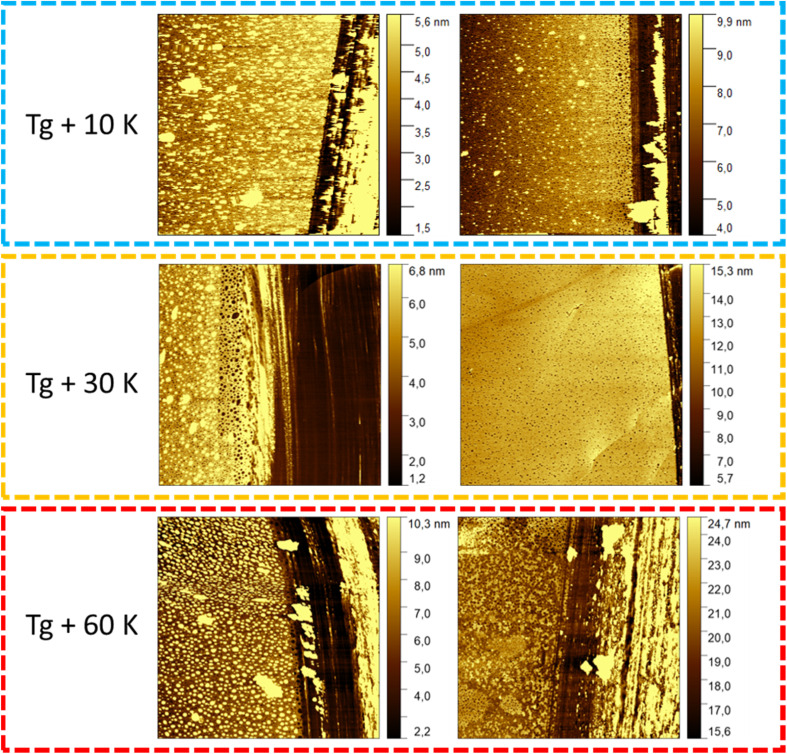
AFM images of the solvent leached samples for the three different annealing temperatures at a shorter (24 h) and longer annealing time (120 h).

**Fig. 5:**
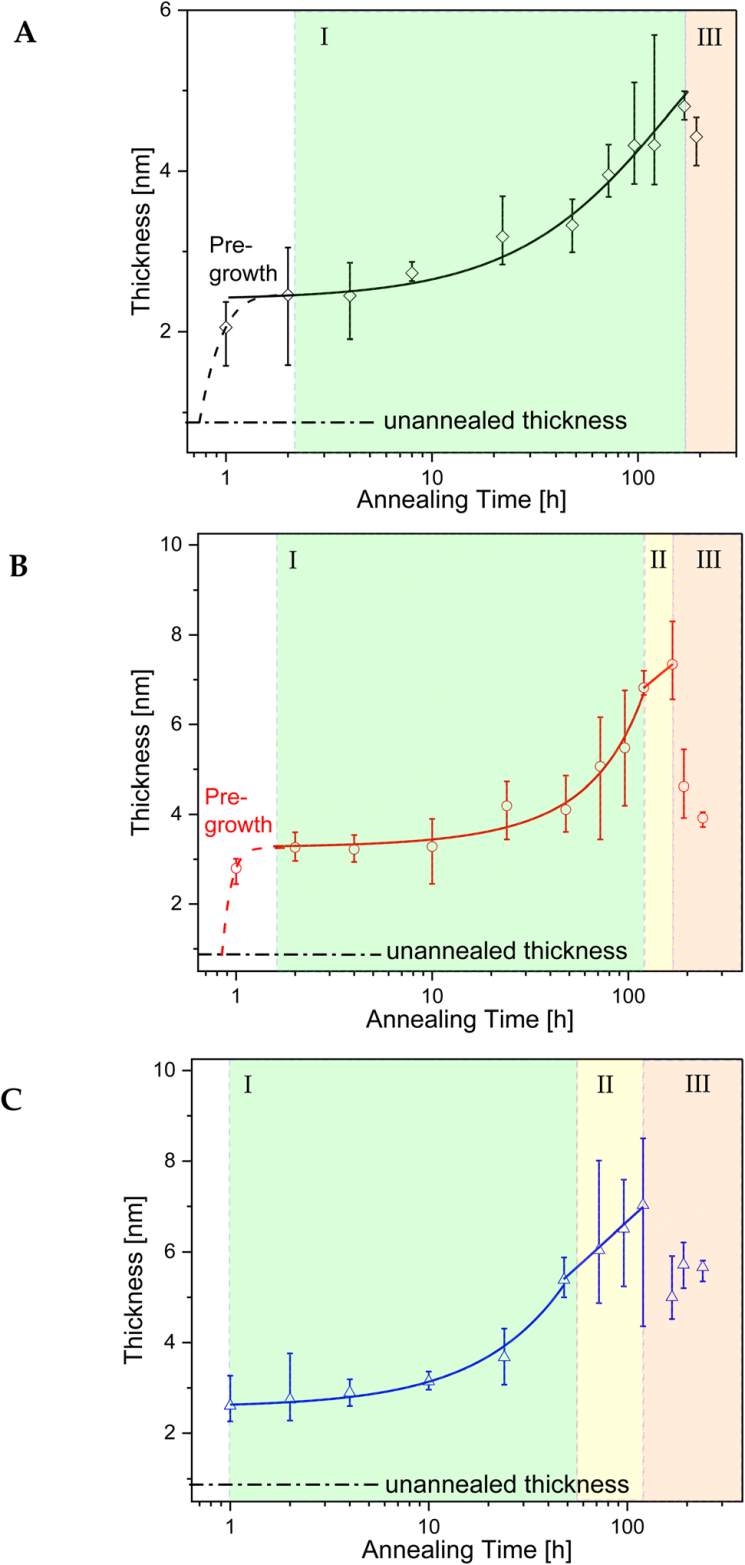
Thickness of the adsorbed layer *versus* annealing time for the three annealing temperatures: (A) *T*_g_ +10 K (black diamonds), (B) *T*_g_ +30 K (red circles), and (C) *T*_g_ +60 K (blue triangles). The open symbols represent the average of 5 to 10 single measurements. The error bars represent the range of where data points are between 10 and 90% of the measured values. The black dot-dashed line represents the thickness of the adsorbed layer of the unannealed sample.

The growth kinetics of the adsorbed layer of PBAC were investigated using the solvent leaching technique, described above. Three annealing temperatures above the glass transition temperature of bulk PBAC value were chosen (*T*_g_ +10 K, *T*_g_ +30 K, and *T*_g_ +60 K) where various annealing times from 1 to ∼240 h were employed. Fig. 4, shows the topography image of the adsorbed layer obtained from the AFM measurements for two annealing times (24 h, and over 120 h) for the three annealing temperatures. The only sample that showed signs of dewetting was the sample annealed at *T*_g_ + 60 K for times longer than 120 h. The thickness of the adsorbed layer was obtained as an average of 5 to 10 different measurements. The thickness of the adsorbed layer is plotted *versus* annealing time for the three annealing temperatures in Fig. 5.

**Fig. 6:**
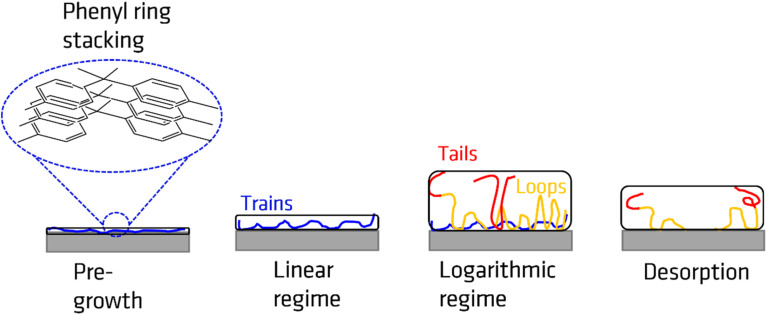
Schematics of the adsorbed layer formed in the different regimes.

The growth kinetics of the adsorbed layer of PBAC has a complicated time dependence with a multi-step time dependence. For all annealing temperatures the thickness of the annealed adsorbed layer is larger than for the unannealed case. This step is denoted as pre-growth region which was not observed for more simple polymers such as polystyrene^30,65^ or P2VP.^66^ Therefore, it is assumed that the pre-growth region is caused by the main chain structure of PBAC. The thickness of the adsorbed layer for the unannealed sample was found to be less than 1 nm (0.8 to 0.9 nm). Chemical model calculations for benzene dimers show that by π–π interaction the most stable configuration of this dimers is a slip-parallel orientation of the benzene rings.^67^ The interaction energy is −2.48 kcal mol^−1^ where the distance of the dimers is *ca.* 0.37 nm. Assuming that this model calculation will have been also relevance for the phenyl rings in PBAC it can be concluded that the phenyl groups in the backbone of PBAC can have a preferential parallel orientation to the surface of the substrate. The initial state of the spin-coating process might be responsible for the formation of a thin adsorbed layer with a thickness of 0.8 to 0.9 nm, where the phenyl rings from dimers have a preferential parallel orientation to the surface of the substrate. These preferentially oriented dimers can be considered as a kind of nucleus that further phenyl rings will stack on top of each other. The pre-growth step observed for PBAC is therefore assigned to the formation of stacks of phenyl rings in the early stages of the annealing process. A schematic cartoon of the pre-growth regime is given in Fig. 6.

Fig. 5 shows further that the pre-growth depends on temperature. With increasing temperature, the pre-growth process becomes faster as the molecular mobility of the segments increases with temperature and so the phenyl groups can stack faster with increasing temperature.

Previous other studies reported a two-step growth process of the adsorbed layer^28,30^ where the two regimes can be modelled through the following equations:

2 *h*_ads_ (*t* < *t*_c_) = *h*_0_ + *νt*

3
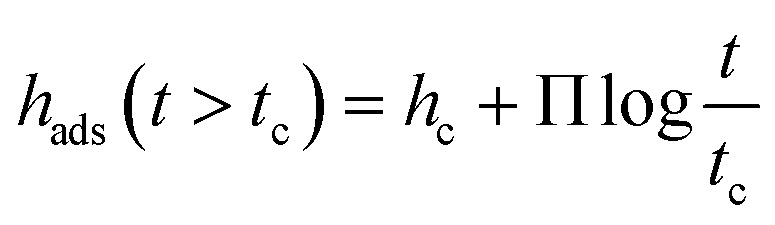
where *h*_ads_ is the thickness of the adsorbed layer, *h*_0_ is the thickness unannealed layer after pre-growth step, *ν* is the growth rate in the linear regime, Π is a parameter characterizing the growth in the logarithmic regime. *t*_c_ is the crossover time from the linear to the logarithmic regime where *h*_c_ is the thickness of the adsorbed layer at *t*_c_. For the adsorbed layer annealed at *T*_g_ +10 K, after the pre-growth region a linear growth regime is observed where the segments are pinned directly to the substrate forming mainly trains (labelled as region I). A schematic of the adsorbed layer formed in the linear regime showing trains is depicted in Fig. 6. Due to the relatively low annealing temperature no crossover of the linear growth mechanism to a logarithmic one is observed in the experimentally covered time window. For the annealing temperature *T*_g_ + 30 K an indication of a cross-over to a logarithmic grow is observed at a time of 120 h where for the annealing temperature of *T*_g_ + 60 K the crossover to the logarithmic growth is detected with a *t*_c_ of 24 h (region II). For the logarithmic growth the segments must diffuse through the existing layer and form loops, segments attached at two points, and tails, dangling chain ends.^33^ The structure of the adsorbed layer formed in the logarithmic growth regime consisting of loops and tails is given in Fig. 6.

**Fig. 7:**
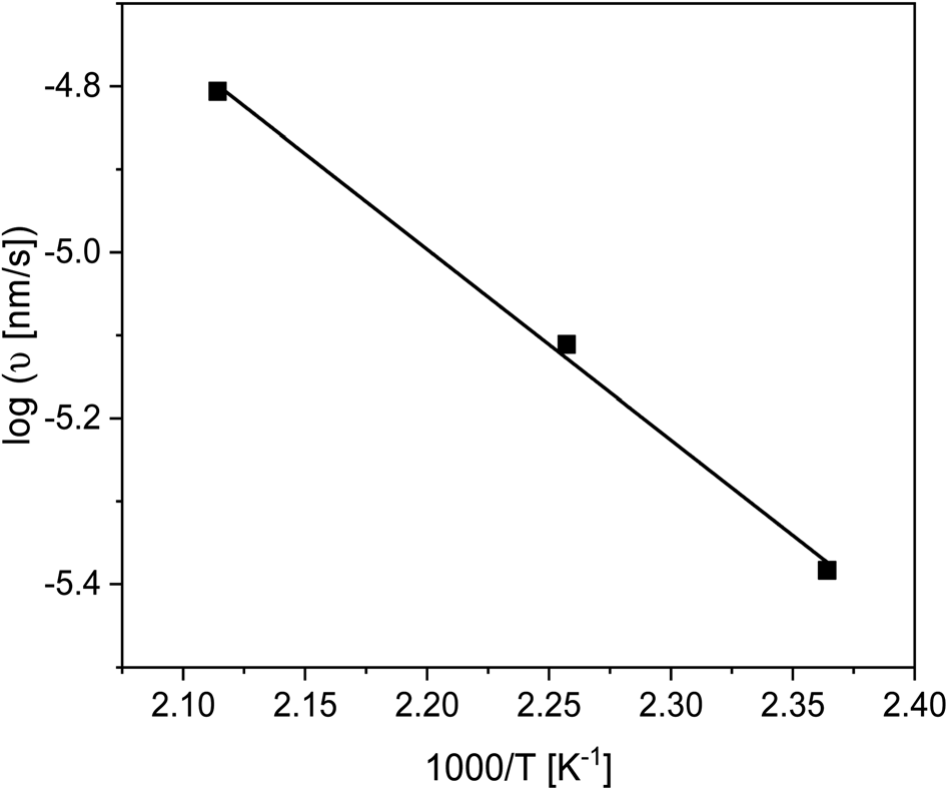
Growth rate in the linear regime *versus* inverse annealing temperature. The solid line is a linear regression to the data.

The linear growth rate *ν* was estimated using eqn (2) and plotted as a function of inverse annealing temperature in Fig. 7. The growth rate increases with increasing temperature, as expected. From the slope of the log(*ν*) *versus* 1000/*T* the activation energy through the Arrhenius equation can be estimated to be 44 kJ mol^−1^. Surprisingly this activation energy is lower than those found for polystyrene^30^ and P2VP.^66^

**Fig. 8:**
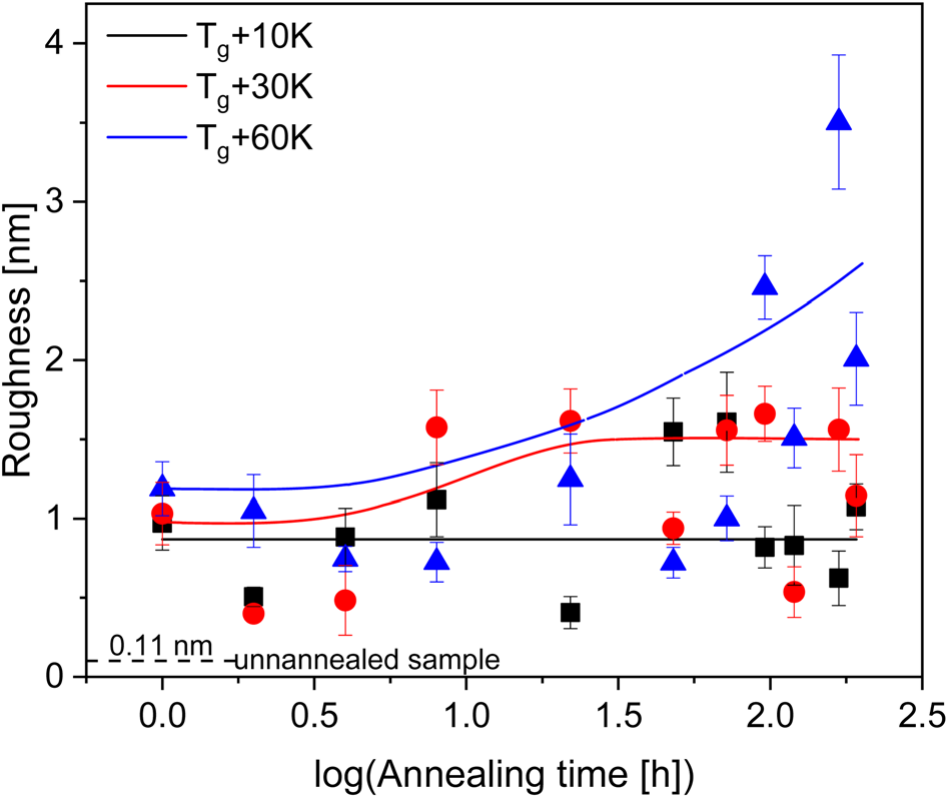
Averaged surface roughness for the adsorbed layers. The error bars represent the uncertainty in the measurements. The dashed line gives the surface roughness of the unannealed sample.

For all annealing temperatures at annealing times longer than 168 h the thickness of the adsorbed layer decreases, illustrated in Fig. 5 as the red zone (region III). This directly contradicts what has been previously reported where the layer thickness stays constant or increases at the same rate in its respective regime.^28,30,33,55,65,66^ This can be discussed as an indication of desorption where segments are simultaneously detaching from the substrate due to the prolonged annealing at high temperatures (see Fig. 6). For the highest annealing temperatures, the highest degree of desorption is observed. As the polymer chains cannot leave the substrate this desorption process will lead to a dewetting of the film. Some indication for the dewetting is already observed in the AFM images for the longest annealing time at the highest temperature, as mentioned above (see Fig. 4). This is further investigated by considering the averaged surface roughness. These values were obtained by measuring the averaged roughness in a truncated window of 5 μm by 5 μm close to the scratch through surface. Additionally, any larger artifacts, which originated from the scratch process causing film to be displaced, were excluded from the averaging process. The surface roughness of each sample was estimated for all leached films and is plotted as a function of the annealing time for the three annealing temperatures, see Fig. 8. For the unannealed sample the surface roughness was quite low with a value of 0.11 nm indicating a smooth adsorbed layer surface. Due to the higher surface roughness for the annealed samples, it is concluded that its increase is a temperature induced effect. The surface roughness for the samples annealed at *T*_g_ + 10 K showed low values for surface roughness (around 1 nm) and was almost independent of the annealing time. For the annealing temperature *T*_g_ + 30 K, the surface roughness is increased, compared to that at the lower annealing temperature. For short annealing times the surface roughness is constant until a step-like increase was observed to a value of *ca.* 1.5 nm at longest annealing times. For the highest annealing temperature, the surface roughness is the highest and increases strongly with annealing time. These results can be discussed in the framework of pre-dewetting or dewetting processes (for the highest annealing temperature and longest annealing times) causing chain desorption in the adsorbed layer. It was discussed previously in literature that dewetting can be correlated to the chain motions in the adsorbed layer after annealing.^68^

## Conclusion

The glass transition behavior of thin films of PBAC were studied employing ellipsometry. The *T*_g_ increased by 25 K compared to the bulk value with the reduction in film thickness down to 28 nm. This was attributed to the formation of an adsorbed layer with reduced mobility compared to bulk PBAC. Unfortunately, for thin films with a thickness below 28 nm the glass transition cannot be estimated because the ellipsometric signals could not be modelled due to the uncertainty of the measurement and PBAC having quite low optical parameters.

The growth kinetics of the adsorbed layer of PBAC was studied by leaching samples from a 200 nm thin film for several annealing times at three different annealing temperatures (*T*_g_ + 10 K, *T*_g_ + 30 K, and *T*_g_ + 60 K). The thickness of the obtained adsorbed layers was measured by AFM employing multiple scans. Additionally, an unannealed sample was measured and the comparison of the unannealed and the annealed sample provided a proof of a pre-growth regime for all annealing temperatures. This pre-growth regime is assigned to the formation of stacks of phenyl groups. For the lowest annealing temperature, after the pre-growth regime, the thickness of the adsorbed layer increases linearly with time where the mechanism of growth is a direct pinning of segments to the surface. For this annealing temperature no crossover to a logarithmic growth regime was observed. The samples annealed at higher temperatures showed a crossover from a linear growth regime to logarithmic growth after some time, 120 h and 24 h for *T*_g_ + 30 K and *T*_g_ + 60 K, respectively. For the longest annealing times the adsorbed layers showed signs of dewetting where segments of the adsorbed films are being detached from the substrate. The measured surface roughness also increased at long annealing times for both *T*_g_ + 30 K and *T*_g_ + 60 K, indicating that chain desorption does occur. The work reported here will be extended by considering other mainchain polymers like polysulfone.

## Conflicts of interest

The authors have no conflicts of interest.

## Supplementary Material

RA-013-D3RA02020G-s001
