# Supplementary material for: Growth kinetics of the adsorbed layer of poly(bisphenol A carbonate) and its effect on the glass transition behavior in thin films
Source: RSC Adv. 2023 May 11;13(21):14473–83. doi: 10.1039/d3ra02020g (PMC10173819; doi:10.1039/d3ra02020g)
Supplement: RA-013-D3RA02020G-s001 [file RA-013-D3RA02020G-s001.pdf]

Electronic Supplementary Material (ESI) for RSC Advances.
This journal is © The Royal Society of Chemistry 2023

Supporting Information

Growth Kinetics of the Adsorbed Layer of Poly (bisphenol-A carbonate) and its Effect on the Glass Transition Behavior in Thin Films

*Hassan Omar, Gundula Hide, Paulina Szymoniak, Andreas Hertwig and Andreas Schönhals**

Bundesanstalt für Materialforschung und –prüfung (BAM),

Unter den Eichen 87, 12205 Berlin (Germany)

*CORRESPONDING AUTHOR: A. Schönhals, BAM Bundesanstalt für Materialforschung und -prüfung (Fachbereich 6.6), Unter den Eichen 87, 12205 Berlin, Germany;
Tel. +49 30 / 8104-3384; Fax: +49 30 / 8104-1617; Email: Andreas.Schoenhals@bam.de

Analysis of the ellipsometric data:

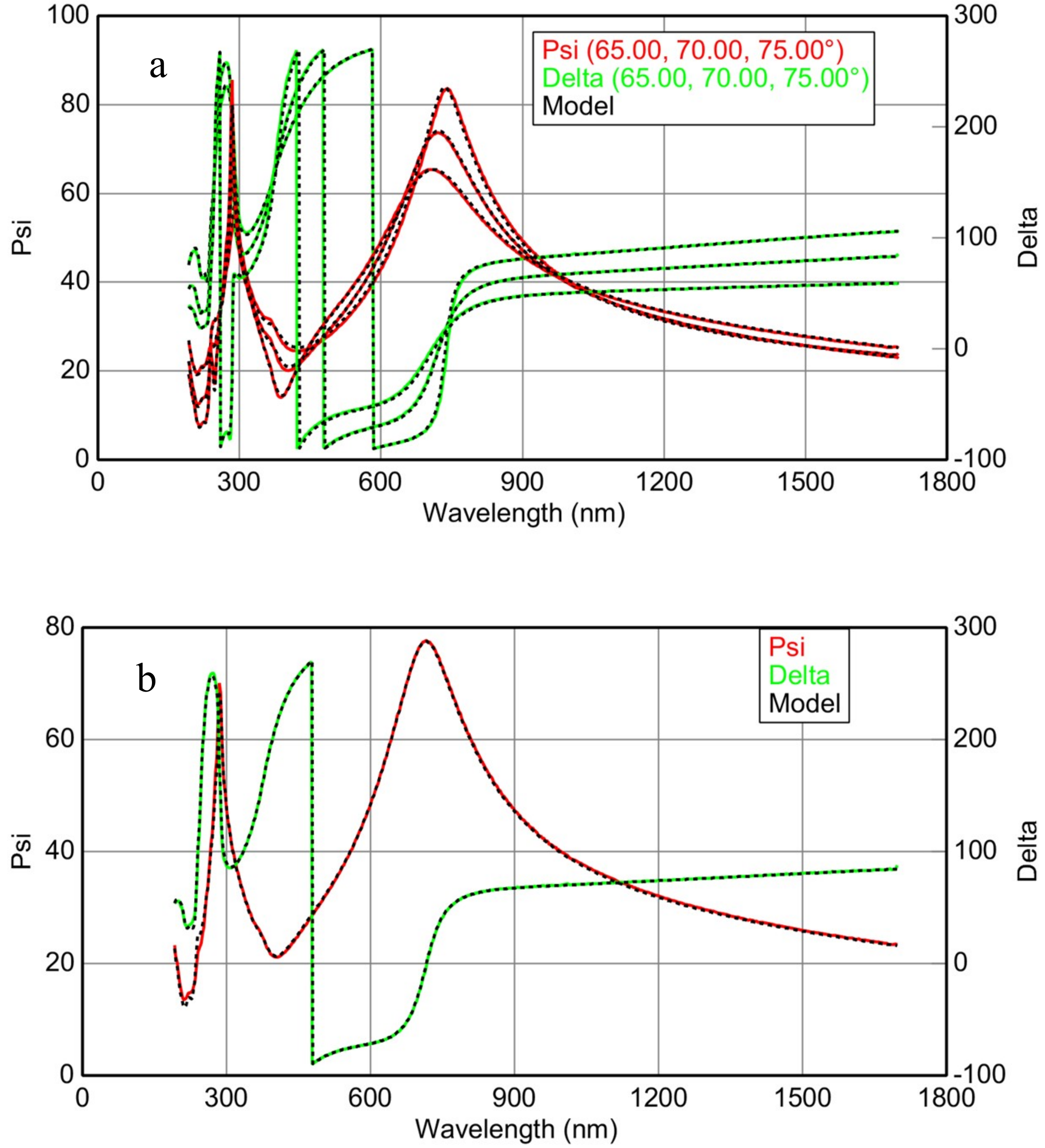

Figure S1: Examples for the analysis of the ellipsometric data (Psi - red, delta – green) for a PBAC film with a thickness of 140 nm**:** a – multi-angle measurements at the angles 65 °, 70 ° and 75 °, b – Single angle measurement at 70 ° during a heating scan at 180 °C (t=62 min). The dotted lines in both figures are the fit of the model to both set of data.

Comparison of the film thickness obtained by AFM measurements and spectroscopic ellipsometry:

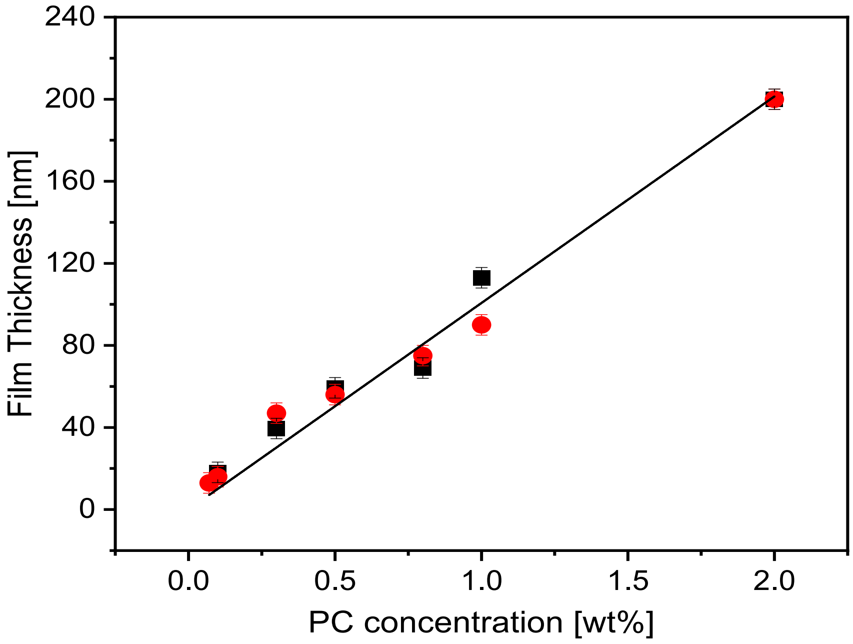

Figure S2: Film thickness of various diluted solutions measured by both AFM (black squares) and Ellipsometry (red circles). The solid line is a linear fit to both sets of data

Analysis of the DSC measurements:

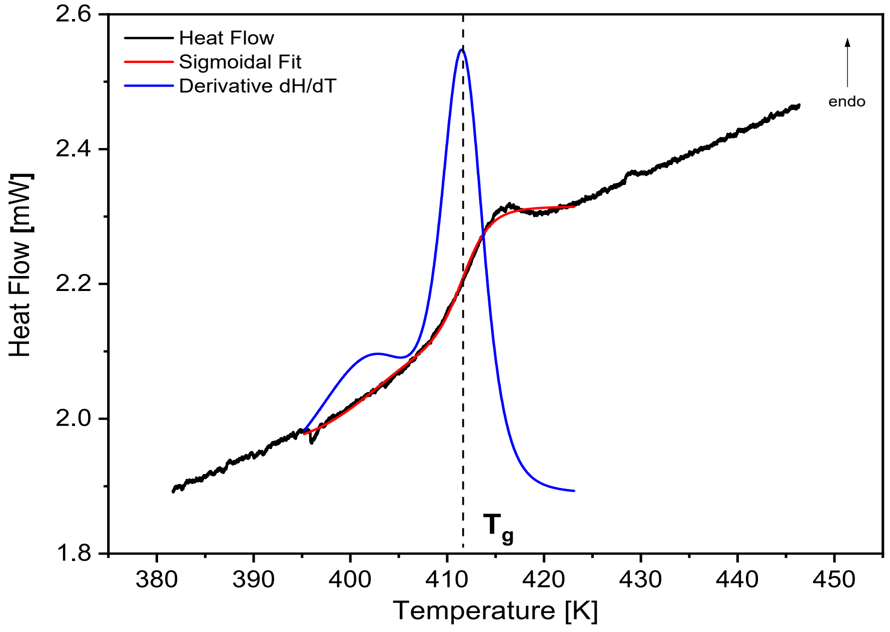

Figure S3: Heat flow curve (black line) with a sigmoidal function fit to the data (red line). The derivative of the sigmoidal fit (blue line) with the peak height taken as the glass transition temperature (T_g).
